# Chronic encapsulated mediastinal abscess presenting with remote cutaneous fistulization 12 years after redo aortic valve replacement for prosthetic valve endocarditis

**DOI:** 10.1186/1749-8090-1-22

**Published:** 2006-08-24

**Authors:** Pankaj Kaul, Syed SA Qadri, Mohd Riaz

**Affiliations:** 1 Leeds General Infirmary, Great George Street, Leeds, LS1 3EX, UK

## Abstract

Chronic encapsulated mediastinal abscess is an unusual complication of previous open heart surgery. We report on the case of a 79 year old male who presented with epigastric fistulization of an encapsulated anterior mediastinal abscess 12 years after a redo aortic valve replacement for prosthetic valve endocarditis. The encapsulated abscess and its complex branching tracts and the cutaneous fistula were excised completely except the thin longitudinal strip of the ascending aorta which formed part of the posterior wall of the infected tract. This was covered with transposed greater omentum based on right gastroepiploic artery pedicle. Patient remains fit and well 2 years after his operation.

This report is unusual on account of the length of the interval between previous heart surgery and the infective complication, the presumed dormancy of the abscess for as long as 12 years, the complex course, branching tracts and the contents of the abscess, the remote fistulization of the abscess at a distant anatomical site and, finally, the principle of successfully covering an infected tract which formed the adventia of the ascending aorta with pedicled omentum in the hope of avoiding an ascending aortic replacement in a frail 79 year old man.

In the entire English language literature, this report represents the longest interval between a heart operation and a sternal or mediastinal abscess

## Background

Mediastinitis is a well known complication of open heart surgery. Chronic mediastinal abscess presenting years after the original heart operation is a medical curiosity. We report the unusual case of a chronic encapsulated mediastinal abscess presenting with cutaneous fistulization in the epigastrium 12 years following redo aortic valve replacement for prosthetic valve endocarditis

## Case report

**Figure 1:**
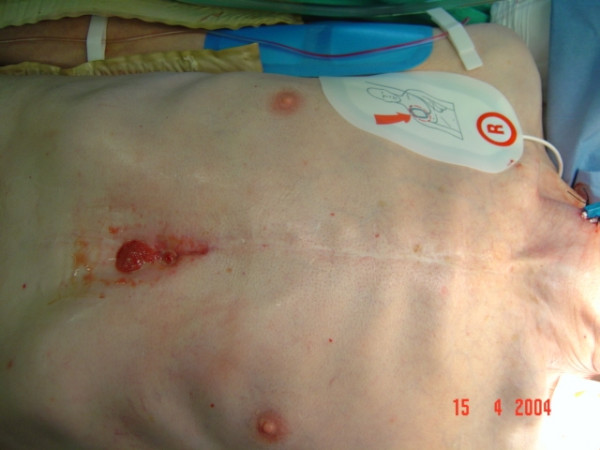
Anterior mediastinal abscess presenting as an epigastric swelling with sinus.

**Figure 2:**
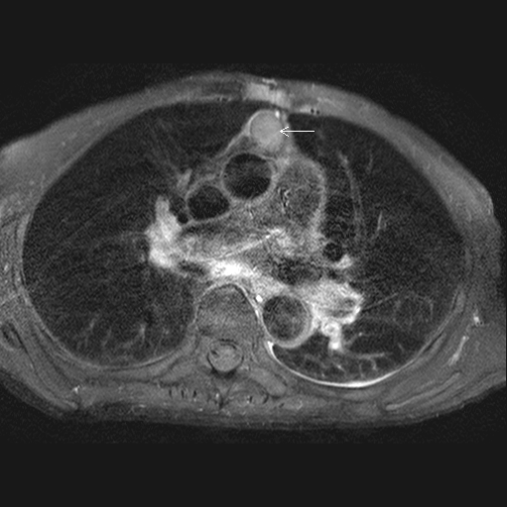
MR scan showing encapsulated mediastinal abscess (arrow).

**Figure 3:**
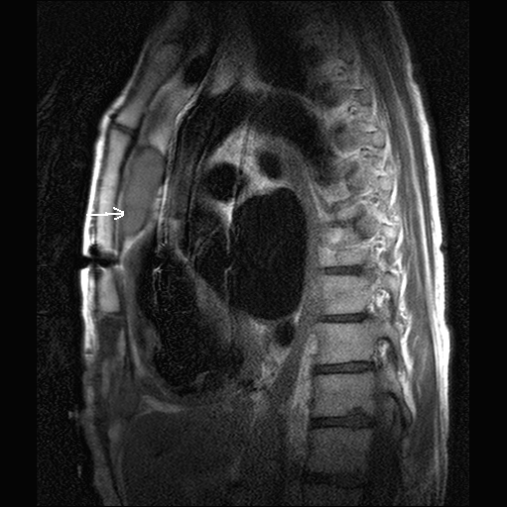
MR scan showing anterior mediastinal abscess in close relation to aorta (arrow).

A 79 year old man presented with a discharging sinus in the epigastrium. He had undergone a mechanical aortic valve replacement for aortic stenosis 14 years ago which was followed 2 years later by repeat mechanical aortic valve replacement for streptococcal prosthetic valve endocarditis. His postoperative recovery following his second valve replacement was uneventful. He continued to be well and asymptomatic with a well healed sternal wound for the next 12 years. However, 12 years following his 2^nd ^operation, he presented with a discharging sinus in epigastrium (fig 1) which was initially treated with dressings and antibiotics by his GP. Exploration of the sinus under general anaesthetic was undertaken after the sinus failed to heal. The sinus was in communication with anterior mediastinum by penetrating the rectus sheath and was divided at the point of entry into mediastinum and removed. All sternal wires were removed. Microbiology of the excised tissue revealed staphylococcus aureus infection and this was treated with appropriate antibiotics for 4 weeks. However, the wound failed to heal although he never developed any signs of systemic illness. A CT scan of chest showed an encapsulated mass extending from ascending aorta to the diaphragm and normal sternum. Transthoracic echocardiography ruled out pseudoaneurysm of ascending aorta, prosthetic endocarditis or aortic root abscess. An MR scan confirmed a longitudinal retrosternal mass extending from mid ascending aorta to the diaphragm (figs 2 and 3) and cutaneous fistulization to the epigastrium through left rectus sheath.

**Figure 4:**
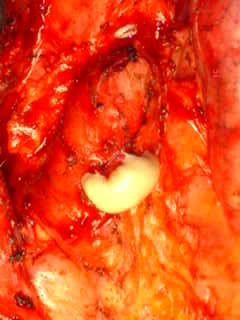
Thick white cheesy material coming out of the mass after aspiration with a thick bore needle.

**Figure 5:**
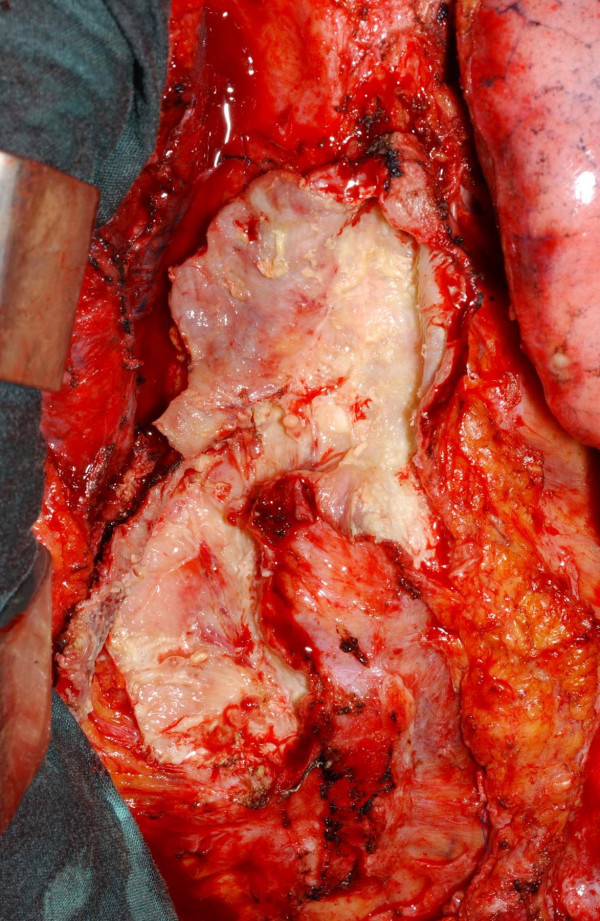
The abscess cavity with all its branching tracts laid open.

**Figure 6:**
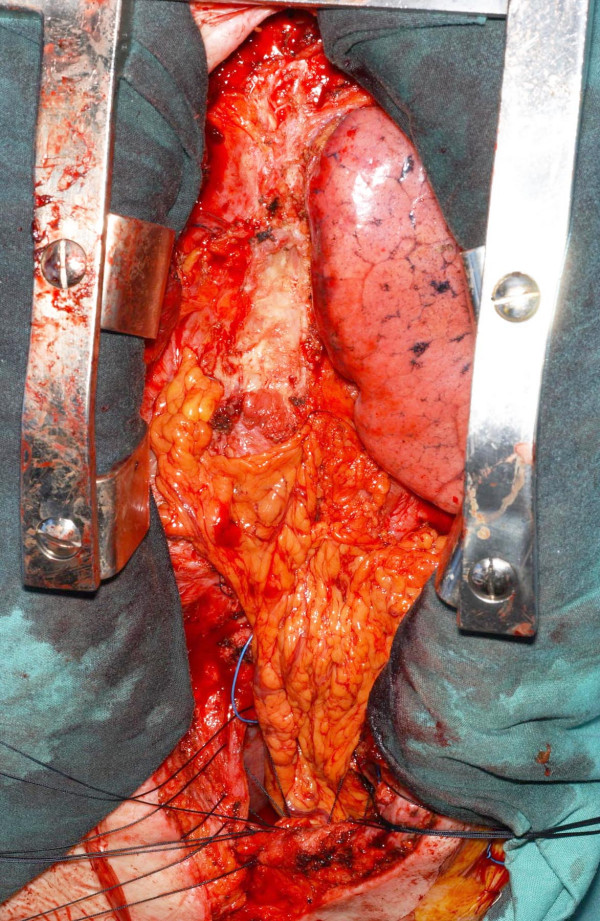
Transposed greater omentum used to cover the thin residual strip of the abscess cavity.

An exploratory secondary median sternotomy was made with cardiopulmonary bypass on standby. Heart and the great vessels were dissected out within the pericardial cavity. A nonpulsatile longitudinal 15 cm mass was found on the anterior surface of ascending aorta and right ventricle along the entire length of anterior mediastinum. At the level of the diaphragm, the mass exited the mediastinum by penetrating the left rectus sheath and communicated with the skin in the epigastrium. At the junction of the superior 1/3 and inferior 2/3, there was a branching tract extending from the main tract to the right and inferiorly over right atrium and the hilum of right lung. The upper part of the mass was aspirated with a wide bore needle when thick cheesy material came out (fig 4). The entire mass and the branching tract were laid open (fig 5), only the middle 2 inches of the mass over the right ventricle having no lumen. The superior half of the tract that lay open was, interestingly, filled with old suture material and Teflon pledgets in addition to thick cheesy material mentioned before. The inferior part of the tract, that had fistulized through the rectus sheath to the epigastric skin, was filled with yellow fresh pus and a piece of old temporary pacing wire. Both the white cheesy material and the pus were sent separately for bacteriological analysis and both grew staphylococcus aureus. The upper mass was almost completely excised except for a thin rim posteriorly which was densely adherent to aorta and the adjacent right atrium and right ventricle. The lower mass was completely excised along with the contiguous portions of rectus muscle, xiphoid, subcutaneous tissue, the sinus and the core of the epigastric skin. Through the same incision, the diaphragm and peritoneum were opened. The greater omentum, based on the right gastroepiploic pedicle, was freed from the greater curvature of the stomach, divided to the far left of the stomach and transposed into the chest to cover the entire raw area of the excised mass and sinus and secured in place with stitches (fig 6). Patient received intravenous antibiotics for 6 weeks and was discharge home thereafter, his sternal wound having healed satisfactorily. At follow up 2 years later, he remains fit and well.

## Discussion

Serious sternal wound infection and dehiscence occurred in 1.86% of a study group of 2579 consecutive cardiac surgical procedures reported by Ottino et al [1]. Stable et al noted a 1.5% incidence of sternal infection, mediastinitis and dehiscence amongst the 13,285 cardiac procedures that he studied [2]. The CABG group had 1.7% and the valve group 0.7% incidence. In a five year audit, Upton reported 0.8%–2.3% post sternotomy mediastinitis with 79% cases caused by staphylococci [3]. There are hardly any reports in English language literature of the incidence of postoperative mediastinitis after cardiac surgery in the absence of sternal wound infection or dehiscence. This is partly owing to the difficulty of obtaining a sample of mediastinal fluid in the presence of an intact sternum after the chest drains have come out. Theoretically, at least, one must assume there is a possibility that the anterior medistinal tissues could get infected while the overlying bone and soft tissues did not. Such a situation would obtain while operating on obviously contaminated tissues as for instance in endocarditis. Mediastinitis would then declare itself, acutely, either with systemic bacterial invasion and/or infected sternal dehiscence or, later and much more rarely, with a more circumscribed anterior mediastinal abscess [4].

What is unique about our patient is the 12 year dormancy of the abscess, absence of any clinical signs during this period and the eventual cutaneous fistulization remotely into the epigastrium. In search of the entire English language literature, this represents, to our knowledge, the largest interval between open heart surgery and a mediastinal or sternal infective complication. Mediastinal abscess, declaring itself after as long as 5 years after CABG, as remote complication of a retained pacing wire fragment, has been reported [5].

Cutaneous fistulization of a recent mediastinal abscess from descending necrotising mediastinitis with a cervical fistula has also been described [6]. It is likely that in our patient, epigastric fistulization of the complex abscess with meandering branching tracts took place in a preformed tract formed by the retained pacing wire fragment.

Staphylococcal infection, in contradistinction to streptococcal infection, is known to predispose to the formation of encapsulated and localised infections. A particularly low grade infection could stay dormant for a long period of time. Another possibility to be kept in mind is a transient bacteraemia of unknown origin giving rise to a metastatic abscess in an old haematoma.

Although the inferior part of the abscess was completely removed, a small posterior longitudinal strip of the organised abscess cavity could not be removed as that would have mandated a complete ascending aortic replacement. This residual strip was covered by greater omentum transposed from abdomen on the right gastroepiploic pedicle. Postoperative infections of ascending aorta and transverse arch have been treated in a variety of ways including the use of viable omentum and muscle flaps [7-10]. Sternal spaces have been filled with rectus abdominis [11,12], latissimus dorsi [13], trapezius [14], pectoralis major [15] and omental flaps [16-18]. There is evidence that omentum contains large number of immunologically active cells that accounts for its anti infective properties [19]. We used the transposed omental pedicle in preference to pectoralis or any other muscle flap because of a number of reasons. First, geographically, the transposed omentum covered the whole of the space occupied by the excised abscess cavity from the mid aorta to the exit point in the left rectus. No other muscle flap, with the possible exception of a rectus abdominis flap, would have achieved this and the rectus flap wouldn't have been substantial enough. Secondly, since the sternum was healthy and was sought to be closed primarily and since it was the retrosternal dead space that was sought to be filled, any other muscle flap, except the rectus abdominis, would have interfered with the primary closure of the sternum. Also, the vascularity, the sheer spread and elasticity of the omentum were felt to be distinct advantages particularly when covering an obviously infected space.

Other pathologies that may present as anterior mediastinal masses postoperatively are mediastinal chylomas, saphenous vein graft aneurysms, ruptured pseudo aneurysms of aorta etc.

To conclude, mediastinal infections can masquerade in a variety of ways. Cutaneous fistulization of a chronic encapsulated abscess can happen years after surgery and requires complete excision of the abscess cavity and the fistulous tract and obliteration of the dead space by omental or muscle flaps.
